# Affordable Nutrient Density in Brazil: Nutrient Profiling in Relation to Food Cost and NOVA Category Assignments

**DOI:** 10.3390/nu14204256

**Published:** 2022-10-12

**Authors:** Alfonso Mendoza-Velázquez, Jonathan Lara-Arévalo, Kennya Beatriz Siqueira, Mariano Guzmán-Rodríguez, Adam Drewnowski

**Affiliations:** 1 Center for Public Health Nutrition, University of Washington, Seattle, WA 98195, USA; 2 Centro de Investigación e Inteligencia Económica, Universidad Popular Autónoma del Estado de Puebla (UPAEP), Puebla 72410, Mexico; 3 Embrapa Dairy Cattle, Juiz de Fora 36038-520, Minas Gerais, Brazil

**Keywords:** Nutrient Rich Food Index, nutrient density, food price, affordability, priority micronutrients, NOVA classification, Brazil

## Abstract

Affordable nutrient density is provided by low-cost and nutrient-rich foods. We explored nutrient density, cost, and NOVA category assignments within and across food groups in Brazil. The nutrient density of the foods (*n* = 591) was assessed using the Nutrient Rich Food Index (NRF9.3) based on protein, fiber, vitamin A (RAE), vitamin C, vitamin E (mg), Ca, Fe, K and Mg; and NRF6.3 score for priority nutrients: Ca, Fe, Zn, vitamin A, vitamin B12, and folate. Nutrients to limit (LIM) were saturated fat, added sugar, and sodium. Affordability was defined as the ratio of energy and/or nutrient density of foods and retail price per 100 kcal. Foods were classified as minimally processed (*n* = 106), processed (*n* = 188), ultra-processed (*n* = 286), and culinary ingredients (*n* = 11). Nutrient density was positively linked to per 100 kcal food cost. Ultra-processed foods (UPF) contained more energy, fat, sugar, and salt and had lower NRF scores compared to minimally processed (MPF) foods. UPF was also less expensive than MPF foods. Nutrient-rich foods below the median per 100 kcal costs included MPF foods, but also processed foods (PF) and UPF. Affordable nutrient-rich foods can be found in the different categories of the NOVA classification.

## 1. Introduction

Affordable nutrient density has been the theme of reports by the Food and Agriculture Organization of the United Nations (FAO) [1,2]. A nutrient-rich diet is essential to improve pregnancy outcomes [3], promote optimal growth and development in childhood [4], and reduce the risk of non-communicable diseases in later life [5]. Around 2 billion people suffer from micronutrient deficiencies worldwide, with populations of low- and middle-income countries (LMIC) known to be affected the most [1,6]. Traditional LMIC diets built around starchy staples often lack micronutrients that are essential for optimal health. Many starch-oriented diets can also lack high-quality protein [7].

Among the priority micronutrients for LMIC are vitamin A, calcium, iron, zinc, folate, and vitamin B12 [6]. Past studies of global food composition databases [6,8,9] have identified the top food sources of priority micronutrients as organ meats, small fish and shellfish, canned fish with bones, dark green leafy vegetables, eggs, milk, and beef, lamb, mutton, and goat. Cheese, goat milk, and pork were also reported to be good sources of priority micronutrients, and so were fresh fish, yogurt, and dry beans [6,8]. While these foods fall into the minimally processed category in the NOVA classification system [10], they can vary considerably in price. First, based on FAO’s own reports, healthy diets worldwide can cost up to five times as much as those built around starches and grain crops [2]. Second, there will be a price differential between, e.g., organ meats, beef and seafood. Third, not all nutrient-rich foods are universally acceptable; some good or excellent nutrient sources may not conform to local or regional social norms [11].

The present goal was to address the relationship between food nutrient density and food cost in Brazil, using a nutrient composition database and national food prices. Based on FAO food balance sheets for 2013 [12], the nutrient composition of the Brazilian diet was 1242 kcal/day from cereals, pulses, and starchy roots (38.1%) and a further 1006 kcal/day (30.8%) from sugars, oils, and fats. Many of those foods may have been prepared at home. Other studies [13] have estimated that the Brazilian diet is composed of 58.1% unprocessed or minimally processed foods, 10.6% processed foods, 20.4% ultra-processed foods and 10.9% culinary ingredients. The consumption of foods falling into the ultra-processed category was associated with a higher intake of added sugars and saturated fat and with lower consumption of protein, fiber, vitamins, and minerals. In another study, the low price of ultra-processed foods per kilogram was associated with a higher prevalence of obesity and overweight, mainly among the lower socioeconomic groups [14].

This study used nutrient profiling methods to assess the nutrient density of foods in the Brazil nutrient composition database. The foods were aggregated by food group and by NOVA categories: minimally processed (MPF), processed (PF), ultra-processed (UPF) and culinary ingredients. The nutrient database was merged with national food prices adjusted for preparation and yield. Two versions of the Nutrient Rich Food (NRF) Index nutrient-density score were used to assess the relative healthfulness of foods: the NRF9.3 score and the NRF6.3 score that was based on priority micronutrients: vitamin A, calcium, iron, zinc, folate, and vitamin B12. The goal was to identify those foods or food groups that were both affordable and nutrient-rich.

## 2. Materials and Methods

### 2.1. Nutrient Composition Database

The Brazil Consumption Expenditure Survey (POF) 2008–2009 [15] from the Brazilian Institute of Geography and Statistics (IBGE) was the source used to obtain the nutrient composition data for all the foods commonly consumed in Brazil. The original database includes 1971 foods and beverages, their preparation description, and their nutritional composition data, including calories (kcal) and 36 different macro and micronutrients. The food groups represented in the different products included in the database are cereals and pulses; flours, starches and pasta; fruits, vegetables, nuts, sweets and confectionery, condiments; meat and offal; fish and seafood; canned and preserved foods; poultry and eggs; dairy products; bakery products; processed meats; soft drinks and infusions; alcoholic and other non-alcoholic beverages. The database contains both MPF and packaged PF.

For the present analyses—including the NRF9.3, NRF6.3, and the affordability metric used—complete data of the nutrients of interest was required for each food in the database. While our interest is in the analysis of the most commonly consumed foods in Brazil, several products in the original database were excluded. Many products had different names but identical nutrient composition data. Duplicate nutrient composition data were identified and removed from the database. Excluded from the analyses were also baby foods, herbs, and spices (e.g., saffron, pepper, mint), cappuccino (diet and light), coffee powders, flours, mate, diet candy and chewing gum. Dried herbs, other powders and flour are not typically classified as foods. Low-caloric beverages (energy density < 10 kcal/100 g), non-caloric beverages, diet beverages, tonic water, and plain water were excluded from the analyses. Alcoholic beverages were excluded as well. The nutrient composition was not available for several local foods; therefore, only foods with complete data for the nutrients of interest were included. Some data on added sugar had to be imputed. For some relevant food groups, such as sweetened beverages, cakes, pies, and other sweets, added sugar was the same as total sugar. The final analytical database, after all these adjustments, was composed of 591 unique items, with no missing values, at least 12 nutrients of interest and had national food prices per 100 g and per 100 kcal, adjusted for edible portions.

For ease of comparison to other studies, unique foods in the Brazil Consumption Expenditure Survey (POF) database were aggregated into food groups and food categories using the What We Eat in America (WWEIA) 4-digit categories and the first 2 digits of the 8-digit WWEIA food codes [16]. The 11 WWEIA food groups were vegetables, sugars, snacks and sweets, protein foods, mixed dishes, milk and dairy, grains, fruits, fats and oils, condiments and sauces, and beverages. The 167 WWEIA categories were the same as in previous studies [17].

### 2.2. The NOVA Classification Scheme

The NOVA Food classification system has been used to designate foods into one of four different categories: unprocessed/minimally processed (MPF), processed (PF), ultra-processed (UPF), and culinary ingredients [10]. Edible parts of plants or of animals are part of the MPF category, where fruits, vegetables, eggs, legumes, and unprocessed meats are included. These food groups are typically referred to as the ones that have a higher nutrient density. The culinary ingredients category includes substances obtained from unprocessed foods or from nature using processes such as refining and milling [10]. Examples of this category include salt, sugar and molasses, vegetable oils and butter. PF are foods that include a combination of MPF and culinary ingredients. Foods such as canned vegetables, fruits and legumes, salted nuts and seeds, cheeses, and salted meats are included in this category. On the other hand, ultra-processed foods (UPF) and drinks are industrial formulations, typically with five or more ingredients, that result from various industrial processes [18].

NOVA category assignments in the present analysis followed published guidelines and were carried out and verified by two researchers. Unprocessed or MPF foods are defined as those that do not include added sugar, added sodium, or added oils and fats [10]. To this category were assigned fruits, vegetables, grains, or meats, fresh, dry, or frozen. Also classed as MPF were fresh milk and plain yogurt, vegetables, freshly squeezed juices, eggs, legumes, fish and other seafood, and unsalted nuts and seeds. The NOVA scheme assigns bread to the MPF category if they are unprocessed, simple, and homemade (by domestic staff). The PF group included simple products made by adding sugar, oil, butter, salt, or vitamins to MPF, as well as vinegars and alcohols. Brazilian mixed dishes, salted nuts and seeds, homemade bread and sauces, cheeses, and salted, cured, and cooked meats were classified as PF. Assigned to the UPF category were commercial (but not homemade) bread, ready-to-eat breakfast cereals, dressings and industrial sauces, bakery and cakes, sweet snacks, pizzas, French fries, ice creams and frozen meals [18,19]. Following prior practice, items such as chicken, beef, vegetables, and grains that were prepared with industrial additives like ketchup, dressings, sauces, or other ingredients were also classified as UPF. Culinary ingredients included salt, sugar and molasses, honey, syrup, vegetable oils, animal fats (butter), additives, preservatives, and starches.

### 2.3. Food Prices Adjusted for Yield and Affordability Metrics

Food prices for 591 food items in Brazilian Reals (R$) per kg or L were obtained from the IBGE Consumption Expenditure Surveys (POF) 2017–2018 and were merged with the nutrient composition database. Food prices were projected using monthly inflation rates from the IBGE Extended Consumer Price Index (IPCA) through December 2021. That is, individual food prices of January 2018 were factored for each food by monthly inflation rates in every period until December 2021. Following standard convention [20], food prices were also yield-adjusted for preparation and waste by dividing each food price by the corresponding yield following the United States Department of Agriculture (USDA) and food guides in Brazil [21] to provide food prices in Brazilian Reals per 100 kcal, edible portion, for each food in the database. The IBGE surveys are used to update consumer price indexes and purchasing power of salaries per city in Brazil. The methods to calculate the National Consumer Price Index (IPCA) for Brazil and more details on the price collection and IPCA methodology can be found elsewhere [22]. Our measure of affordability results from dividing nutrient scores by the cost of foods. This objective measure ignores other factors taken into account by consumers when choosing one food over the other such as disposable income, inflation, individual wealth, and physical barriers, among other social barriers [23]. However, this notion of affordability is a lower bound, a necessary but insufficient condition for food security [1].

### 2.4. Nutrient Density 

#### The Nutrient Rich Food Approach

This study employs nutrient-rich food (NRF) scores [24] to assess the nutritional quality of foods. NRF scores are useful for discriminating between nutrient-rich foods from energy-dense but nutrient-poor foods [24,25]. These scores comprise several beneficial nutrient density components to encourage (NRn) and some nutrients to limit (LIMk), such as saturated fat, added sugar, and sodium [25]. The NRFn.k score subtracts from the *n* beneficial nutrients to include (*Nut_inc_i_*) the *k* nutrients to limit (*Nut_lim_j_*) as follows:

(1) $$NRn=\left(\frac{Nut\_inc_{1}}{DV_{1}}+\frac{Nut\_inc_{2}}{DV_{2}}+\ldots +\frac{Nut\_inc_{2}}{DV_{n}}\right)\times \left(100/ED\right)$$

(2) $$LIMk=\left(\frac{Nut\_\lim_{1}\;}{MRV_{1}}+\ldots +\frac{Nut\_\lim_{k}}{MRV_{k}}\right)\times \left(100/ED\right)$$

(3) $$NRFn.k=\left[\overset{n}{\underset{i=1}{\sum }}\frac{Nut_{\_}inc_{i}}{\left(Dv_{i}\right)}-\overset{k}{\underset{j=1}{\sum }}\frac{Nut\_\lim_{k}\;\;}{\left(MRV_{j}\right)}\right]\times \left(100/ED\right)$$

standardized with energy density (ED), i.e., kcal per 100 g. The NRF9.3 model was based on nine nutrients to encourage (protein, fiber, vitamin A (RAE), vitamin C, vitamin E (mg), calcium, iron, potassium and magnesium) and three nutrients to limit (LIM) (saturated fat, added sugar and sodium). The NRF6.3 priority nutrients model was based on six nutrients to encourage (calcium, iron, zinc, vitamin A, vitamin B12 and folate) and three nutrients to limit (saturated fat, added sugar and sodium). Although the present scoring NRF6.3 algorithm was very different from that used by Ortenzi and Beal [6], it was based on the same six priority nutrients [6,8]. The NRF9.3 score was the same as used in many previous studies. Two versions of the LIM sub-score were used: one based on added sugar (LIM) and the other based on total sugar (LIMt).

Nutrient standards followed the recommendations of the Brazilian Ministry of Health [26] as well as the standards adopted by Codex Alimentarius [27]. Since the Brazilian diet presents significant nutrient gaps in vitamin E [28], vitamin E was included in the NRF9.3 algorithm. Reference daily value (DVi) for each nutrient was set at protein (50 g), fiber (25 g), vitamin A (800 Retinol Activity Equivalents—RAE), vitamin C (100 mg), vitamin E (9 mg), Ca (1000 mg), Fe (14 mg), K (3500 mg) and Mg (310 mg). The NRF6.3 score employs the following priority nutrients: calcium, iron, zinc, vitamin A, vitamin B12, and folate. The DVi values for vitamin B12 (2.4 mcg), for zinc (11 mg) and folate (400 mcg) [27]. The maximum recommended values (MRVj) for nutrients to limit are saturated fat (20 g), added sugar (50 g) and sodium (2000 mg) [27]. To avoid the over-representation of dietary adequacy, the nutrient percent daily value was capped at 100%.

### 2.5. Plan of Analysis

Differences between variable means by NOVA category assignments were tested using one-way ANOVAs. This procedure provides an F and *p*-value statistic for the differences among the NOVA categories. The statistical package used was Stata MP v.17, Stata Corp, College Station, TX, USA.

## 3. Results

### 3.1. Distribution of Foods by Food Group and NOVA Category

**Figure 1:**
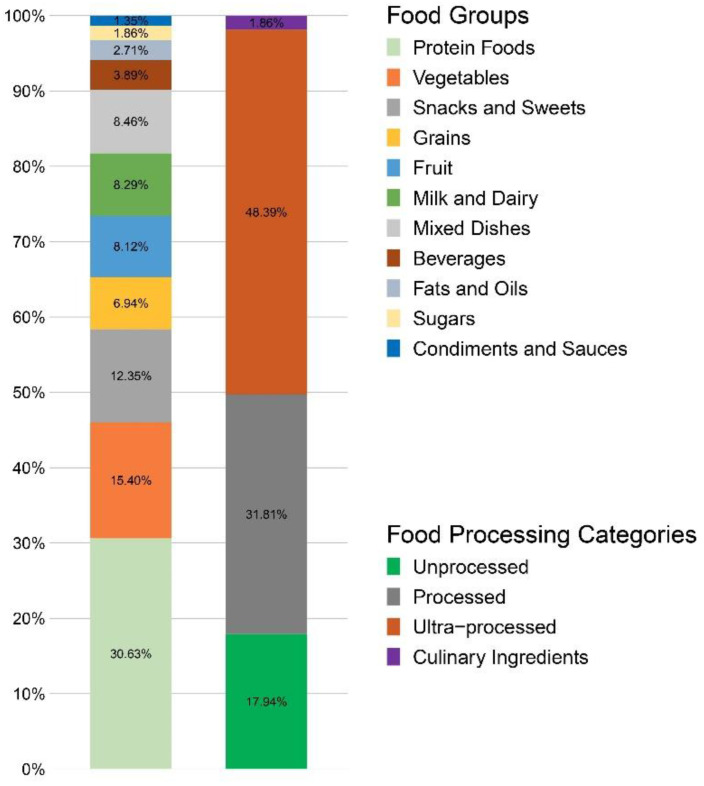
Distribution of foods by food group and NOVA classification system.

Food groups were based on the What We Eat in America (WWEIA) aggregation codes. In the Brazil database, protein foods represented 30.63% of the total and included both animal-based protein foods (meats, poultry, seafood, and eggs) and plant-based protein foods (beans, peas, legumes, nuts and seeds, processed soy products). The other food groups were vegetables (15.40%), snacks and sweets (12.35%), grains (6.94%), fruits (8.12%), milk and dairy (8.29%), mixed dishes (8.46%), beverages (3.89%), fats and oils (2.71%), sugars (1.86%), and condiments and other foods (1.35%). The NOVA assignments were MPF (17.94%), PF (31.81%), UPF (48.39%), and culinary ingredients (1.86%). Figure 1 shows the percentage distribution of foods by food group and NOVA category assignment.

### 3.2. NOVA Categories by Food Group

**Figure 2:**
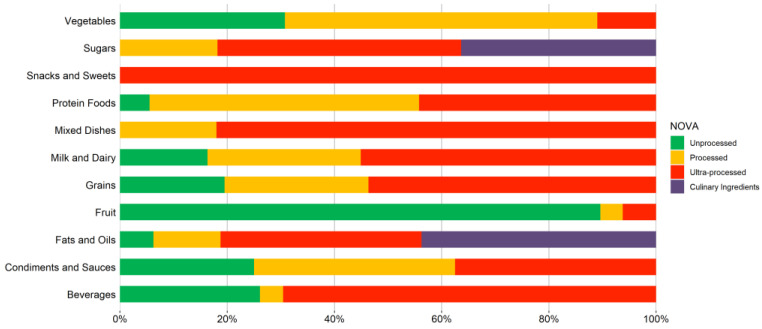
NOVA categories by food group.

Figure 2 shows which food groups were classified as MPF versus UPF. Fruits (89.58%) were most likely to be classified as MPF. The database contained several cooked vegetables (i.e., boiled broccoli with salt) and meats (i.e., steamed chicken with salt) that were prepared with culinary ingredients. Therefore, vegetables (58.24%) and protein foods (50.28%) were most likely to be PF. Snacks (100%), mixed foods (82.00%), beverages (69.57%), milk and dairy (55.10%), and grains (53.66%) were most likely to be classified as UPF. Most of the foods classed as mixed foods were prepared using industrial sauces (i.e., meat dishes prepared with barbeque sauce) or included UPF foods (i.e., soup containing ham) which classified them as UPF. The fats and sugars groups had the most culinary ingredients (43.75% and 36.36%, respectively).

### 3.3. Nutrient Density Scores by Food Group

**Figure 3:**
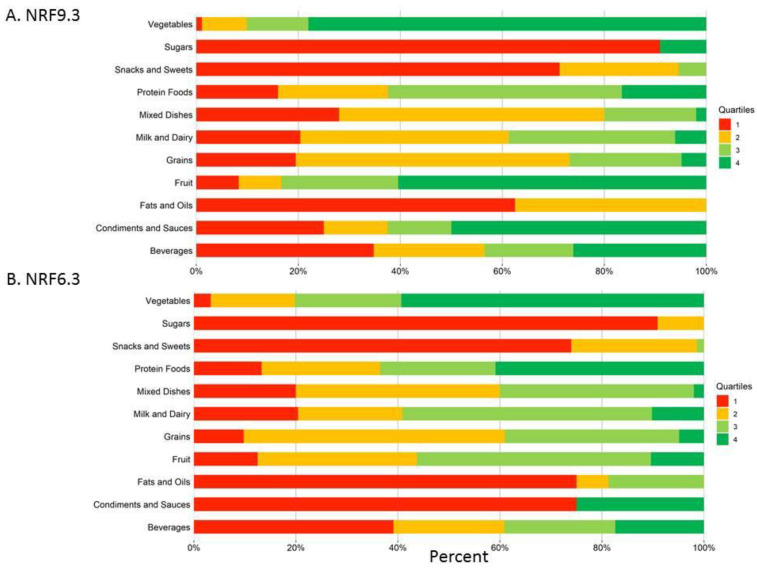
Percent distribution of quartiles of nutrient density scores for each food group. (**A**) Percent distribution of quartiles of nutrient density scores using NRF9.3; (**B**) Percent distribution of quartiles of nutrient density scores using NRF6.3.

Figure 3 shows percent quartile distributions of nutrient density scores for each food group. Found in the top quartile of NR9.3 scores (Figure 3A) were vegetables (78.02%), fruits (60.42%), condiments (50.00%) and beverages (26.09%). Found in the bottom quartile were sugars (90.91%), snacks (71.23%), fats (62.50%) and beverages (34.78%). Found in the top quartile of NR6.3 scores (Figure 3B) were vegetables (59.34%), proteins (40.88%), condiments (25.00%), and some beverages (17.39%), milk (12.24%) and fruits (10.42%). Found in the bottom quartile were sugars (90.91%), snacks (72.60%), fats (75.00%), condiments and sauces (75.00%) and beverages (39.13%).

Table 1 shows that MPF had the highest NR9 scores (127.0) and the lowest LIM and LIMt scores (6.9 and 17.1). UPF had higher LIM (25.4) and LIMt (22.3) scores. Culinary ingredients (sugar, oils) had the lowest NR9 scores (10.3) and the highest LIM (34.7) and LIMt (26.0) scores. PF had the highest NR6 scores (76.9). UPF and culinary ingredients had the lowest NR6 scores (35.4 and 5.4, respectively).

**Figure 4:**
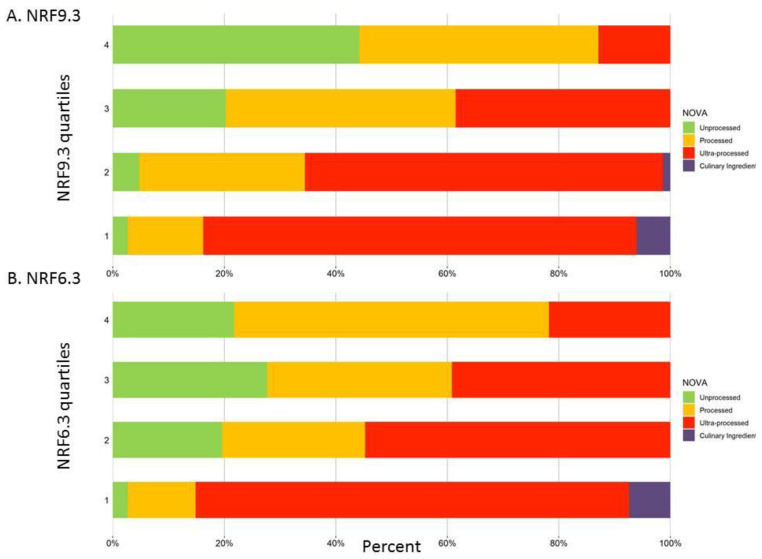
Nutrient density scores distribution by NOVA categories. (**A**) NRF9.3 quartile distribution by NOVA categories; (**B**) NRF6.3 quartile distribution by NOVA categories.

Figure 4 shows the overlap between NOVA categories and nutrient density scores. Shown are the percent distribution of quartiles of nutrient density scores for each NOVA food category. Figure 4A shows that foods in the top quartile of NRF9.3 scores were mostly MPF (44.52%) as opposed to foods categorized as UPF (13.01%). By contrast, foods in the bottom quartile of NRF9.3 scores were mostly UPF foods (77.70%) and PF (13.51%). Figure 4B shows that foods in the top quartile of NRF6.3 scores were mostly PF (56.08%), UPF (22.30%) and MPF (21.62%). Foods in the bottom quartile of NRF6.3 scores were mostly UPF foods (77.55%), followed by PF (12.24%) and culinary ingredients (7.48%), as opposed to MPF (2.72%). While most foods classified as UPF were in the bottom quartiles of nutrient density scores, the top quartiles of both NRF9.3 and NRF6.3 scores did include some foods classified as UPF (13.01% and 22.30%).

### 3.4. Cost by Nutrient Density and NOVA Categories

Table 2 shows that foods assigned to the UPF category had higher energy density and contained more saturated fat, total sugar and added sugar, as compared to other NOVA categories. MPF had the lowest energy density (125.2 kcal/100 g), followed by PF (160.3 kcal/100 g), UPF (255.5 kcal/100 g), and culinary ingredients (603.5 kcal/ 100 g). UPF and culinary ingredients had the lowest cost per 100 kcal (R$1.3 and R$0.5, respectively). MPF had the highest cost per 100 kcal (R$3.1).

**Figure 5:**
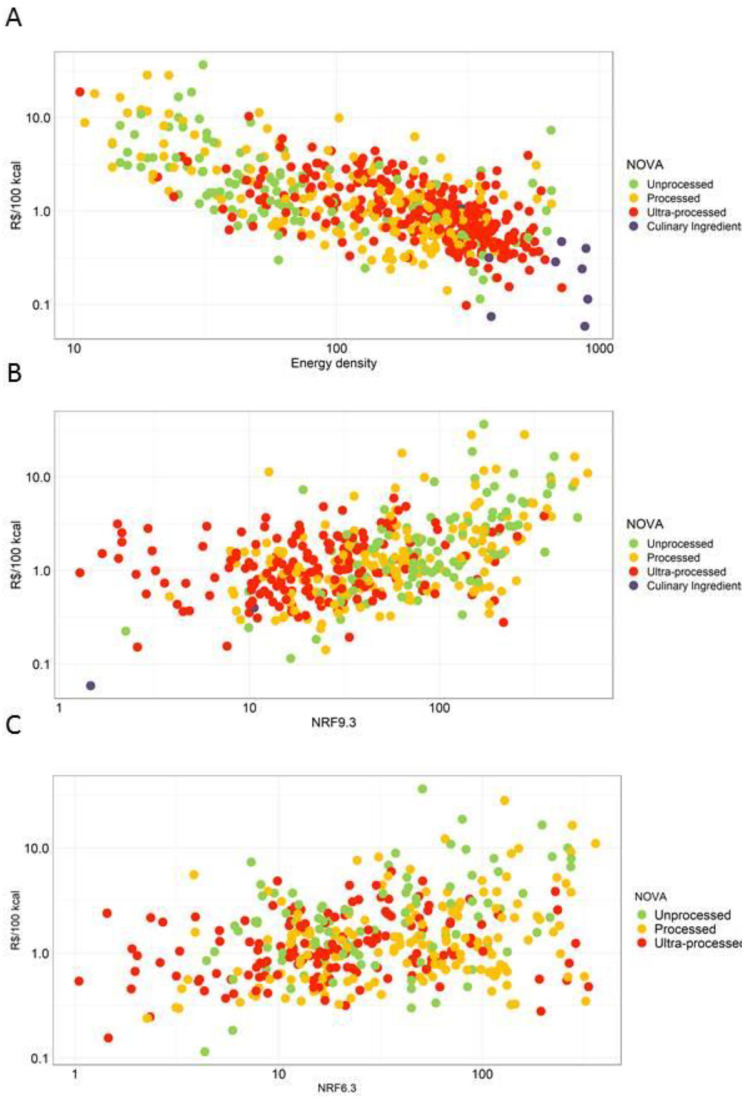
Price by NOVA categories and nutrient density. (**A**) Price by NOVA categories and energy density; (**B**) Price by NOVA categories and NRF9.3; (**C**) Price by NOVA categories and NRF6.3.

Figure 5 panels also indicate NOVA category assignments. Figure 5A shows the expected relation between higher energy density (kcal/g) and lower per kcal cost (R$/100 kcal (*r* = −0.3473). Figure 5B shows the positive correlation between higher NRF9.3 scores and higher per kcal cost (*r* = 0.4293). Figure 5C shows the positive correlation between higher NRF6.3 scores and higher per kcal cost (*r* = 0.2202). As had been noted before, foods in the UPF category tended to be energy dense, had lower nutrient density scores and lower per kcal costs. However, as indicated in the Figure 5 panels, there were some UPF foods that were both inexpensive and nutrient-rich. Some of those foods provided the needed priority nutrients, as captured by the NRF6.3 score.

### 3.5. Affordable Nutrient Density: High NRF Scores at below Median Cost

Table 3 shows the UPF foods in the highest two quartiles of positive nutrient density (NRF9.3) and below the median price in $R per 100 kcal. Among the foods categorized by the NOVA system as UPF, some chicken parts, liver and organ meats, nutritional beverages, white potatoes, milk substitutes, starchy vegetables, and nuts can be found due to the inclusion of ultra-processed sauces or condiments in their preparations. For example, chicken liver stewed with regular tomato sauce costs R$0.48 per 100 kcal, less than 84% of the foods in the database.

## 4. Discussion

The present analyses evaluated the nutrient density of foods in Brazil using two separate nutrient density metrics. The NRF9.3 nutrient density score, based on nine nutrients to encourage and three nutrients to limit, had been used in many previous studies [29,30,31,32]. The new NRF6.3 score was based on six priority nutrients to encourage and the same three nutrients to limit. In prior work [5,33,34], those six nutrients (calcium, vitamin A, iron, zinc, folate, and vitamin B12) were identified as being in short supply in LMIC diets.

The present study was unique in that both national food prices and the NOVA categorization were included in the analyses of food nutrient density and food prices, both calculated on a per 100 kcal basis. The NOVA system divides foods into MPF, PF, UPF and culinary ingredients. First, analyses of NOVA categories by food group showed that about 48% of all foods in the Brazil database were classified as UPF. However, there was much variability among food groups. Whereas fruit was 90% MPF, snacks and sweets were classified as 100% UPF. Foods categorized as UPF were much cheaper (R$ per 100 kcal) than were MPF, mostly fresh meats, vegetables, and fruit. On a per-calorie basis, culinary ingredients (sugar, oils) were the least expensive of all.

MPF had higher NRF9.3 and NRF6.3 nutrient density scores. Consistent with published analyses [6,17,24], both metrics gave higher values to organ meats, lean meats, and poultry and to vegetables and fruits. The study used Brazil’s national food prices adjusted for inflation and corrected for preparation and waste. In general, higher nutrient density scores were associated with higher per 100 kcal food costs. A positive relation between different nutrient density scores and retail prices had also been observed before in Brazil and Mexico [17,35], confirming prior reports from the US [24], France [36] and other high-income countries.

The present data provide new insights into the relationship between NOVA categorization, nutrient density metrics, and the foods’ energy and nutrient cost. Although MPF had higher nutrient density, they cost more per calorie than foods in the PF and UPF categories. Although foods classified as UPF cost less per calorie, many such foods were of lower nutritional value. In general, foods classified as UPF were higher in energy, contained more added fat, sugar and salt, and had lower nutrient density scores overall. Snacks and sweets, grains, beverages, condiments, sugars, cereals, and mixed foods were classed as UPF, which is consistent with other reports [17]. However, there were exceptions since a strict application of the NOVA criteria also captured milk and dairy, proteins, vegetables, and fruits. Some of these foods were given high nutrient-density scores.

The present analyses allowed us to identify foods in the highest quartiles of NRF9.3 score and below the median of the cost that could be described as both affordable and nutrient-rich. The main question was whether all such foods would be MPF or would that group also include some foods in the PF and UPF categories. Our analyses showed that many foods that provided affordable nutrient density were classed as UPF by the NOVA system. Among these were chicken liver, beef liver pate, cereals, cocoa, fish, and seafood, all of which had been prepared with added industrial ingredients and therefore fell into the UPF category.

Other studies have also pointed to a conflict between high nutrient density ratings and UPF category assignments [37,38,39]. We now provide further evidence—for Brazil—that some nutrient-rich foods appear to fall into the category of UPF. While confirming that UPF foods generally do contain more fat, sugar, and sodium and can be low in micronutrients [13,17,19], the present analyses point to other foods that are likely misclassified using the published NOVA criteria.

The purpose of this study was to explore affordable nutrient density in relation to the NOVA categories, using foods and food prices in Brazil. Local studies suggest that for the last 30 years, MPF in Brazil has been replaced by foods in the UPF category [40]. It is now estimated that about 20% of total dietary energy in Brazil comes from these food products [40], still well below the estimated 58% for the United States [41]. The low price of foods in the UPF category has been remarked on before; it has been associated with a higher prevalence of overweight and obesity, mostly among lower socioeconomic groups [14]. Those authors suggest that foods in the UPF category should be taxed to reduce the prevalence of obesity.

However, based on the current classification system, the UPF category did include fortified foods such as bread and well as beverages, which can be a source of essential vitamins and minerals [42]. The present analyses used a new score based on LMIC priority nutrients, previously identified by Beal et al. [6,8]. Calcium, vitamin A, iron, zinc, folate, and vitamin B12 are often lacking in diets of low- and middle-income countries. Often found in organ meats, seafood and shellfish, leafy greens and other MPF, those nutrients can also be found in fortified industrial products. It does not help that the NOVA system assigns foods with priority nutrients to the UPF category.

As shown by modeling studies, nutritionally adequate diets can be composed of foods from the four NOVA categories [37]; it is important not to exclude any foods that can be both low-cost and nutrient-rich. One economic interpretation of the finding that lower income groups select foods in the UPF category is that their food purchases are driven primarily by food price. The transition towards higher consumption of UPFs in Brazil [15,16] may be driven by economic status or other variables. Whereas most recommendations have focused on increasing the production of fruits, vegetables, and small-scale livestock [17,18], the role of the PF industry in assuring affordable nutrient density should not be forgotten.

The study had limitations. While the sample of foods was bigger than in previous studies, some tropical fruit and other local foods were missing nutrient data. The present focus on the Brazil nutrient composition data and Brazil food prices may prevent generalization to the other LMICs. For example, the price of fresh fruit in Brazil was much lower than might have been expected based on data from other countries. The study also used unit value prices derived from consumption and expenditure surveys, which do not necessarily reflect local prices or true food expenditures. That could lead to a bias in the results. Nonetheless, the method of attaching food prices to the nutrient composition database, effectively as another nutrient vector, has been successfully employed and discussed in other studies [24,43], including some previous applications to LMIC and Brazil [17].

## 5. Conclusions

The goal of our study was to determine the relationship between food nutrient density and food cost in Brazil, identifying those foods or food groups that were both affordable and nutrient-rich. The question was whether achieving affordable nutrient density in Brazil can be aided by PF and UPF, as defined by the NOVA system. Nutrient density was positively linked to per 100 kcal food cost. Among foods in the top quartile on nutrient density scores and below the median for food cost, there were foods from three NOVA categories: MPF, PF, and UPF. MPF is associated with higher nutrient density; however, they also have a higher price compared to the other NOVA categories. Affordable nutrient density can be obtained from a wide range of foods, including foods from PF and UPF NOVA categories. Studies on LMIC priority nutrients should include prices and costs to identify foods that are both affordable and nutrient-rich.

## Figures and Tables

**Table 1:** Energy density and nutrient density by NOVA categories.

Food Items	NR6		NR9		LIM		LIMt	
	*n*	Mean (SD)	*n*	Mean (SD)	*n*	Mean (SD)	*n*	Mean (SD)
All items	591	51.3 (61.4)	591	73.9 (87.8)	591	19.9 (18.4)	589	20.7 (16.4)
NOVA categories								
Minimally processed	106	53.6 (65.5)	106	127.0 (108.6)	106	6.9 (14.1)	104	17.1 (14.4)
Processed	188	76.9 (70.9)	188	98.5 (103.9)	188	18.0 (21.7)	188	20.1 (22.3)
Ultra-Processed	286	35.4 (46.3)	286	40.4 (42.5)	286	25.4 (14.4)	286	22.3 (11.9)
Culinary ingredients	11	5.4 (6.8)	11	10.3 (9.7)	11	34.7 (17.2)	11	26.0 (12.5)
*p*-Value *		<0.0001		<0.0001		<0.0001		<0.0279

* ANOVA was used to compare the means between NOVA food categories.

**Table 2:** Energy density, nutrient content, and prices per 100 g and per 100 kcal by NOVA categories.

						Price (R$)	
Food Items		Energy Density kcal/100 g	Saturated Fat g	Total Sugar g	Added Sugar g	Per 100 g	100 kcal
	N	Mean (SE)	Mean (SE)	Mean (SE)	Mean (SE)	Mean (SE)	Mean (SE)
All items	591	208.7 (6.6)	3.7 (0.3)	9.4 (0.7)	6.9 (0.7)	2.3 (0.1)	1.9 (0.13)
NOVA categories							
Minimally processed	106	127.1 (14.9)	1.5 (0.4)	6.5 (0.9)	0.01 (0.0)	2.3 (0.5)	3.1 (0.4)
Processed	188	160.3 (8.7)	3.1 (0.3)	2.2 (0.5)	0.9 (0.5)	2.1 (0.2)	2.4 (0.3)
Ultra-processed	286	255.5 (8.4)	4.2 (0.3)	14.4 (1.2)	12.7 (1.2)	2.4 (0.1)	1.3 (0.1)
Culinary ingredients	11	603.5 (78.4)	23.1 (7.8)	29.2 (13.0)	31.0 (13.3)	2.1 (0.4)	0.5 (0.1)
*p*-Value *		<0.0001	<0.0001	<0.0001	<0.0001	<0.7923	<0.0001

* ANOVA was used to compare the means between NOVA food categories.

**Table 3:** Ranking of nutrient-rich foods in the top quartile of nutrient density scores and below the median cost per 100 kcal in Brazil.

							Affordability		
No	IBGE Food ID	Food	Energy Density	$R/100 kcal	NRF9.3	NRF6.3	NRF9.3	NRF6.3	NOVA
1	6700301	Kale, cooked from fresh, boiled, salt used	58.9	0.78	253.5	122.9	32.6	15.8	PF
2	6906501	Fortified diet shake, meal replacement, dry powders (not reconstituted)	335.5	0.28	216.8	193.8	27.9	24.9	UPF
3	7104601	Pork, organ meats, liver, stewed or boiled, salt added	165.0	0.60	211.7	316.9	27.2	40.8	PF
4	7801001	Chicken, liver, stewed or boiled, salt added (80 g) + industrial sauce, tomato, regular (20 g)	138.4	0.48	194.3	332.1	25.0	42.7	UPF
5	6400401	Sweet potato, baked, salt added	90.0	0.43	183.2	111.2	23.6	14.3	PF
6	6400501	Yams (sweet potato), baked, salt added	90.0	0.67	183.2	111.2	23.6	14.3	PF
7	7801001	Chicken, liver, fried	189.7	0.35	182.5	321.4	23.5	41.4	PF
8	6400304	Sweet potato, boiled, salt added	76.0	0.97	172.3	111.2	22.2	14.3	PF
9	6401201	Carrots, cooked from fresh, fried or sauteed, fat used	60.5	0.65	167.0	107.9	21.5	13.9	PF
10	6801801	Orange, fresh	47.0	0.76	160.2	29.8	20.6	3.8	MPF
11	6401201	Carrots, cooked from fresh, stir-fried, butter used, regular, salt added	56.7	0.70	158.2	98.2	20.4	12.6	PF
12	7102501	Beef, liver, stewed or boiled, no salt added (80 g) + industrial sauce, tomato, regular (20 g)	157.6	0.80	157.9	267.1	20.3	34.4	UPF
13	6400501	Yams (sweet potato type), cooked from frozen, salt added	100.0	0.60	152.8	114.2	19.7	14.7	PF
14	7102501	Beef, liver, stir-fried, fat used, salt added	213.7	0.59	150.4	262.7	19.3	33.8	PF
15	7102501	Beef, liver, fried, breaded or batter dipped	229.8	0.55	148.2	259.9	19.1	33.4	UPF
16	6804901	Elderberries, fresh	73.0	0.78	145.3	25.4	18.7	3.3	MPF
17	6501301	Cereal, ready-to-eat, wheat germ	360.0	0.34	131.8	59.0	17.0	7.6	UPF
18	6400908	Sweet potato, boiled, fat used, salt added	116.4	0.69	126.5	84.2	16.3	10.8	PF
19	6802602	Pineapple, fresh	48.0	0.84	119.1	16.2	15.3	2.1	MPF
20	6802202	Tangerine, fresh	53.0	0.95	109.1	24.8	14.0	3.2	MPF
21	7264101	Oyster, cooked from fresh or frozen, stewed, or boiled, salt added	137.0	0.98	106.5	241.6	13.7	31.1	PF
22	7903601	Milk, skim, no fat or fat-free	34.2	1.03	96.4	132.6	12.4	17.1	MPF
23	7273101	Fish and seafood, roe, herring, stewed or boiled, salt added	204.0	0.68	95.5	118.6	12.3	15.3	PF
24	8104001	Pate, pork liver	326.0	0.56	94.6	190.6	12.2	24.5	UPF
25	6401101	Beets, raw	43.0	0.95	91.2	75.9	11.7	9.8	MPF
26	7273101	Fish and seafood, roe, herring, fried, salt added	226.7	0.61	86.1	115.8	11.1	14.9	PF
27	6301204	Beans, fava, cooked from fresh, salt added	85.6	1.01	85.1	30.9	11.0	4.0	PF
28	6502101	Nestum 3 RTE cereal Nestlé	345.8	0.70	84.0	53.0	10.8	6.8	UPF
29	6806001	Sapoti (Tropical fruit)	96.0	0.98	83.7	20.7	10.8	2.7	MPF
30	6900818	Cocoa or hot chocolate, Ovaltine hot cocoa—dry mix	388.0	0.61	81.6	45.2	10.5	5.8	UPF

$R/100 kcal: Brazilian Reals per 100 kcal; NRF: Nutrient Rich Food Index; IBGE: Brazilian Institute of Geography and Statistics; MPF: Minimally Processed Food; PF: Processed Food; UPF: Ultra-processed Food.

## Data Availability

The datasets analyzed for this study can be found on the IBGE’s website: https://www.ibge.gov.br/en/statistics/social/social-protection/25610-pof-2017-2018-pof-en.html?edicao=28913&t=resultados (accessed on 1 April 2022) and https://www.ibge.gov.br/en/statistics/social/social-protection/17387-pof-2008-2009-en.html?edicao=19562&t=resultados (accessed on 1 April 2022).
